# Supplementary material for: Pyruvate Dehydrogenase Kinase 1 inhibition mediated oxidative phosphorylation enhancement in cartilage promotes osteoarthritis progression
Source: BMC Musculoskelet Disord. 2023 Jul 20;24:597. doi: 10.1186/s12891-023-06585-6 (PMC10357736; doi:10.1186/s12891-023-06585-6)
Supplement: Supplementary file 1 — Additional file 1. [file 12891_2023_6585_MOESM1_ESM.zip › KEGG.Gsea.1653622432464/KEGG_NATURAL_KILLER_CELL_MEDIATED_CYTOTOXICITY.html]

Details for gene set KEGG\_NATURAL\_KILLER\_CELL\_MEDIATED\_CYTOTOXICITY[GSEA]

|  || Dataset | 55457\_collapsed\_to\_symbols.cls.cls #OA\_versus\_normal.cls.cls #OA\_versus\_normal\_repos |
| Phenotype | cls.cls#OA\_versus\_normal\_repos |
| Upregulated in class | normal |
| GeneSet | KEGG\_NATURAL\_KILLER\_CELL\_MEDIATED\_CYTOTOXICITY |
| Enrichment Score (ES) | -0.22393768 |
| Normalized Enrichment Score (NES) | -0.9873709 |
| Nominal p-value | 0.46769232 |
| FDR q-value | 0.67455363 |
| FWER p-Value | 1.0 |
Table: GSEA Results Summary

  

Fig 1: Enrichment plot: KEGG\_NATURAL\_KILLER\_CELL\_MEDIATED\_CYTOTOXICITY      
 Profile of the Running ES Score & Positions of GeneSet Members on the Rank Ordered List

  

| SYMBOL | TITLE | RANK IN GENE LIST | RANK METRIC SCORE | RUNNING ES | CORE ENRICHMENT || 1 | VAV1 | vav guanine nucleotide exchange factor 1 [Source:HGNC Symbol;Acc:HGNC:12657] | 601 | 0.622 | -0.0272 | No |
| 2 | IFNAR2 | interferon alpha and beta receptor subunit 2 [Source:HGNC Symbol;Acc:HGNC:5433] | 616 | 0.618 | -0.0086 | No |
| 3 | PLCG2 | phospholipase C gamma 2 [Source:HGNC Symbol;Acc:HGNC:9066] | 725 | 0.588 | 0.0017 | No |
| 4 | MAPK3 | mitogen-activated protein kinase 3 [Source:HGNC Symbol;Acc:HGNC:6877] | 757 | 0.579 | 0.0177 | No |
| 5 | ITGB2 | integrin subunit beta 2 [Source:HGNC Symbol;Acc:HGNC:6155] | 827 | 0.562 | 0.0301 | No |
| 6 | IFNGR2 | interferon gamma receptor 2 [Source:HGNC Symbol;Acc:HGNC:5440] | 861 | 0.552 | 0.0451 | No |
| 7 | IFNA8 | interferon alpha 8 [Source:HGNC Symbol;Acc:HGNC:5429] | 1206 | 0.475 | 0.0333 | No |
| 8 | TNFSF10 | TNF superfamily member 10 [Source:HGNC Symbol;Acc:HGNC:11925] | 1241 | 0.468 | 0.0456 | No |
| 9 | ICAM2 | intercellular adhesion molecule 2 [Source:HGNC Symbol;Acc:HGNC:5345] | 2048 | 0.356 | -0.0061 | No |
| 10 | TYROBP | transmembrane immune signaling adaptor TYROBP [Source:HGNC Symbol;Acc:HGNC:12449] | 2058 | 0.355 | 0.0045 | No |
| 11 | CD48 | CD48 molecule [Source:HGNC Symbol;Acc:HGNC:1683] | 2082 | 0.352 | 0.0139 | No |
| 12 | TNF | tumor necrosis factor [Source:HGNC Symbol;Acc:HGNC:11892] | 2098 | 0.350 | 0.0239 | No |
| 13 | PIK3CB | "phosphatidylinositol-4,5-bisphosphate 3-kinase catalytic subunit beta [Source:HGNC Symbol;Acc:HGNC:8976]" | 2186 | 0.340 | 0.0279 | No |
| 14 | LCK | "LCK proto-oncogene, Src family tyrosine kinase [Source:HGNC Symbol;Acc:HGNC:6524]" | 2274 | 0.329 | 0.0316 | No |
| 15 | SHC1 | SHC adaptor protein 1 [Source:HGNC Symbol;Acc:HGNC:10840] | 2485 | 0.304 | 0.0248 | No |
| 16 | SH2D1A | SH2 domain containing 1A [Source:HGNC Symbol;Acc:HGNC:10820] | 2579 | 0.293 | 0.0269 | No |
| 17 | CD244 | CD244 molecule [Source:HGNC Symbol;Acc:HGNC:18171] | 2591 | 0.291 | 0.0353 | No |
| 18 | FCER1G | Fc fragment of IgE receptor Ig [Source:HGNC Symbol;Acc:HGNC:3611] | 2752 | 0.275 | 0.0315 | No |
| 19 | NFATC1 | nuclear factor of activated T cells 1 [Source:HGNC Symbol;Acc:HGNC:7775] | 3027 | 0.247 | 0.0180 | No |
| 20 | NFATC3 | nuclear factor of activated T cells 3 [Source:HGNC Symbol;Acc:HGNC:7777] | 3079 | 0.242 | 0.0217 | No |
| 21 | CASP3 | caspase 3 [Source:HGNC Symbol;Acc:HGNC:1504] | 3202 | 0.230 | 0.0195 | No |
| 22 | KLRC3 | killer cell lectin like receptor C3 [Source:HGNC Symbol;Acc:HGNC:6376] | 3243 | 0.227 | 0.0236 | No |
| 23 | PTPN6 | protein tyrosine phosphatase non-receptor type 6 [Source:HGNC Symbol;Acc:HGNC:9658] | 3434 | 0.208 | 0.0153 | No |
| 24 | KLRK1 | killer cell lectin like receptor K1 [Source:HGNC Symbol;Acc:HGNC:18788] | 3572 | 0.197 | 0.0109 | No |
| 25 | IFNAR1 | interferon alpha and beta receptor subunit 1 [Source:HGNC Symbol;Acc:HGNC:5432] | 3587 | 0.195 | 0.0160 | No |
| 26 | CSF2 | colony stimulating factor 2 [Source:HGNC Symbol;Acc:HGNC:2434] | 3699 | 0.185 | 0.0132 | No |
| 27 | PRKCA | protein kinase C alpha [Source:HGNC Symbol;Acc:HGNC:9393] | 3728 | 0.183 | 0.0168 | No |
| 28 | PPP3CA | protein phosphatase 3 catalytic subunit alpha [Source:HGNC Symbol;Acc:HGNC:9314] | 3803 | 0.176 | 0.0167 | No |
| 29 | FAS | Fas cell surface death receptor [Source:HGNC Symbol;Acc:HGNC:11920] | 3828 | 0.173 | 0.0203 | No |
| 30 | RAC3 | Rac family small GTPase 3 [Source:HGNC Symbol;Acc:HGNC:9803] | 3849 | 0.171 | 0.0242 | No |
| 31 | PAK1 | p21 (RAC1) activated kinase 1 [Source:HGNC Symbol;Acc:HGNC:8590] | 3861 | 0.169 | 0.0287 | No |
| 32 | LAT | linker for activation of T cells [Source:HGNC Symbol;Acc:HGNC:18874] | 3922 | 0.162 | 0.0292 | No |
| 33 | FASLG | Fas ligand [Source:HGNC Symbol;Acc:HGNC:11936] | 3931 | 0.162 | 0.0337 | No |
| 34 | VAV3 | vav guanine nucleotide exchange factor 3 [Source:HGNC Symbol;Acc:HGNC:12659] | 4082 | 0.148 | 0.0267 | No |
| 35 | SYK | spleen associated tyrosine kinase [Source:HGNC Symbol;Acc:HGNC:11491] | 4084 | 0.147 | 0.0313 | No |
| 36 | IFNA4 | interferon alpha 4 [Source:HGNC Symbol;Acc:HGNC:5425] | 4282 | 0.129 | 0.0200 | No |
| 37 | CD247 | CD247 molecule [Source:HGNC Symbol;Acc:HGNC:1677] | 4309 | 0.128 | 0.0220 | No |
| 38 | FCGR3A | Fc fragment of IgG receptor IIIa [Source:HGNC Symbol;Acc:HGNC:3619] | 4594 | 0.105 | 0.0032 | No |
| 39 | KLRD1 | killer cell lectin like receptor D1 [Source:HGNC Symbol;Acc:HGNC:6378] | 4778 | 0.089 | -0.0083 | No |
| 40 | MICA | MHC class I polypeptide-related sequence A [Source:HGNC Symbol;Acc:HGNC:7090] | 5193 | 0.057 | -0.0389 | No |
| 41 | PPP3CB | protein phosphatase 3 catalytic subunit beta [Source:HGNC Symbol;Acc:HGNC:9315] | 5311 | 0.046 | -0.0466 | No |
| 42 | GRB2 | growth factor receptor bound protein 2 [Source:HGNC Symbol;Acc:HGNC:4566] | 5697 | 0.016 | -0.0762 | No |
| 43 | BID | BH3 interacting domain death agonist [Source:HGNC Symbol;Acc:HGNC:1050] | 5715 | 0.014 | -0.0771 | No |
| 44 | SHC2 | SHC adaptor protein 2 [Source:HGNC Symbol;Acc:HGNC:29869] | 5738 | 0.012 | -0.0784 | No |
| 45 | IFNA2 | interferon alpha 2 [Source:HGNC Symbol;Acc:HGNC:5423] | 5869 | 0.004 | -0.0884 | No |
| 46 | KIR3DL2 | "killer cell immunoglobulin like receptor, three Ig domains and long cytoplasmic tail 2 [Source:HGNC Symbol;Acc:HGNC:6339]" | 5893 | 0.002 | -0.0902 | No |
| 47 | RAC2 | Rac family small GTPase 2 [Source:HGNC Symbol;Acc:HGNC:9802] | 6098 | -0.013 | -0.1057 | No |
| 48 | IFNA21 | interferon alpha 21 [Source:HGNC Symbol;Acc:HGNC:5424] | 6127 | -0.015 | -0.1074 | No |
| 49 | GZMB | granzyme B [Source:HGNC Symbol;Acc:HGNC:4709] | 6132 | -0.015 | -0.1073 | No |
| 50 | RAF1 | "Raf-1 proto-oncogene, serine/threonine kinase [Source:HGNC Symbol;Acc:HGNC:9829]" | 6187 | -0.019 | -0.1109 | No |
| 51 | LCP2 | lymphocyte cytosolic protein 2 [Source:HGNC Symbol;Acc:HGNC:6529] | 6263 | -0.025 | -0.1160 | No |
| 52 | IFNG | interferon gamma [Source:HGNC Symbol;Acc:HGNC:5438] | 6410 | -0.036 | -0.1262 | No |
| 53 | PRKCB | protein kinase C beta [Source:HGNC Symbol;Acc:HGNC:9395] | 6453 | -0.039 | -0.1283 | No |
| 54 | SH3BP2 | SH3 domain binding protein 2 [Source:HGNC Symbol;Acc:HGNC:10825] | 6608 | -0.050 | -0.1387 | No |
| 55 | IFNA10 | interferon alpha 10 [Source:HGNC Symbol;Acc:HGNC:5418] | 6682 | -0.054 | -0.1427 | No |
| 56 | IFNA5 | interferon alpha 5 [Source:HGNC Symbol;Acc:HGNC:5426] | 6755 | -0.059 | -0.1465 | No |
| 57 | KLRC1 | killer cell lectin like receptor C1 [Source:HGNC Symbol;Acc:HGNC:6374] | 6835 | -0.066 | -0.1505 | No |
| 58 | MAPK1 | mitogen-activated protein kinase 1 [Source:HGNC Symbol;Acc:HGNC:6871] | 6853 | -0.068 | -0.1497 | No |
| 59 | ZAP70 | zeta chain of T cell receptor associated protein kinase 70 [Source:HGNC Symbol;Acc:HGNC:12858] | 6962 | -0.077 | -0.1557 | No |
| 60 | PPP3CC | protein phosphatase 3 catalytic subunit gamma [Source:HGNC Symbol;Acc:HGNC:9316] | 7031 | -0.082 | -0.1584 | No |
| 61 | IFNGR1 | interferon gamma receptor 1 [Source:HGNC Symbol;Acc:HGNC:5439] | 7176 | -0.094 | -0.1667 | No |
| 62 | RAC1 | Rac family small GTPase 1 [Source:HGNC Symbol;Acc:HGNC:9801] | 7192 | -0.096 | -0.1648 | No |
| 63 | IFNA6 | interferon alpha 6 [Source:HGNC Symbol;Acc:HGNC:5427] | 7272 | -0.101 | -0.1678 | No |
| 64 | NRAS | "NRAS proto-oncogene, GTPase [Source:HGNC Symbol;Acc:HGNC:7989]" | 7300 | -0.102 | -0.1667 | No |
| 65 | KIR2DL4 | "killer cell immunoglobulin like receptor, two Ig domains and long cytoplasmic tail 4 [Source:HGNC Symbol;Acc:HGNC:6332]" | 7384 | -0.109 | -0.1697 | No |
| 66 | PIK3CD | "phosphatidylinositol-4,5-bisphosphate 3-kinase catalytic subunit delta [Source:HGNC Symbol;Acc:HGNC:8977]" | 7405 | -0.111 | -0.1677 | No |
| 67 | PIK3CA | "phosphatidylinositol-4,5-bisphosphate 3-kinase catalytic subunit alpha [Source:HGNC Symbol;Acc:HGNC:8975]" | 7733 | -0.136 | -0.1889 | No |
| 68 | PIK3CG | "phosphatidylinositol-4,5-bisphosphate 3-kinase catalytic subunit gamma [Source:HGNC Symbol;Acc:HGNC:8978]" | 7953 | -0.152 | -0.2012 | No |
| 69 | HRAS | "HRas proto-oncogene, GTPase [Source:HGNC Symbol;Acc:HGNC:5173]" | 7977 | -0.153 | -0.1982 | No |
| 70 | SOS2 | SOS Ras/Rho guanine nucleotide exchange factor 2 [Source:HGNC Symbol;Acc:HGNC:11188] | 8058 | -0.160 | -0.1993 | No |
| 71 | ARAF | "A-Raf proto-oncogene, serine/threonine kinase [Source:HGNC Symbol;Acc:HGNC:646]" | 8092 | -0.163 | -0.1967 | No |
| 72 | BRAF | "B-Raf proto-oncogene, serine/threonine kinase [Source:HGNC Symbol;Acc:HGNC:1097]" | 8171 | -0.169 | -0.1975 | No |
| 73 | PRF1 | perforin 1 [Source:HGNC Symbol;Acc:HGNC:9360] | 8265 | -0.176 | -0.1991 | No |
| 74 | KIR2DL1 | "killer cell immunoglobulin like receptor, two Ig domains and long cytoplasmic tail 1 [Source:HGNC Symbol;Acc:HGNC:6329]" | 8348 | -0.182 | -0.1998 | No |
| 75 | MICB | MHC class I polypeptide-related sequence B [Source:HGNC Symbol;Acc:HGNC:7091] | 8367 | -0.183 | -0.1953 | No |
| 76 | KIR2DS4 | "killer cell immunoglobulin like receptor, two Ig domains and short cytoplasmic tail 4 [Source:HGNC Symbol;Acc:HGNC:6336]" | 8519 | -0.195 | -0.2009 | No |
| 77 | IFNA7 | interferon alpha 7 [Source:HGNC Symbol;Acc:HGNC:5428] | 8533 | -0.196 | -0.1957 | No |
| 78 | CHP2 | calcineurin like EF-hand protein 2 [Source:HGNC Symbol;Acc:HGNC:24927] | 8573 | -0.199 | -0.1924 | No |
| 79 | PRKCG | protein kinase C gamma [Source:HGNC Symbol;Acc:HGNC:9402] | 8582 | -0.200 | -0.1867 | No |
| 80 | IFNA1 | interferon alpha 1 [Source:HGNC Symbol;Acc:HGNC:5417] | 8640 | -0.204 | -0.1846 | No |
| 81 | PIK3R3 | phosphoinositide-3-kinase regulatory subunit 3 [Source:HGNC Symbol;Acc:HGNC:8981] | 8801 | -0.217 | -0.1902 | No |
| 82 | PIK3R5 | phosphoinositide-3-kinase regulatory subunit 5 [Source:HGNC Symbol;Acc:HGNC:30035] | 8804 | -0.217 | -0.1835 | No |
| 83 | ULBP1 | UL16 binding protein 1 [Source:HGNC Symbol;Acc:HGNC:14893] | 9322 | -0.257 | -0.2157 | Yes |
| 84 | FCGR3B | Fc fragment of IgG receptor IIIb [Source:HGNC Symbol;Acc:HGNC:3620] | 9341 | -0.259 | -0.2089 | Yes |
| 85 | ITGAL | integrin subunit alpha L [Source:HGNC Symbol;Acc:HGNC:6148] | 9526 | -0.276 | -0.2145 | Yes |
| 86 | SHC3 | SHC adaptor protein 3 [Source:HGNC Symbol;Acc:HGNC:18181] | 9608 | -0.284 | -0.2118 | Yes |
| 87 | KIR2DL5A | "killer cell immunoglobulin like receptor, two Ig domains and long cytoplasmic tail 5A [Source:HGNC Symbol;Acc:HGNC:16345]" | 9695 | -0.291 | -0.2093 | Yes |
| 88 | ICAM1 | intercellular adhesion molecule 1 [Source:HGNC Symbol;Acc:HGNC:5344] | 9740 | -0.296 | -0.2033 | Yes |
| 89 | MAP2K2 | mitogen-activated protein kinase kinase 2 [Source:HGNC Symbol;Acc:HGNC:6842] | 9932 | -0.315 | -0.2082 | Yes |
| 90 | TNFRSF10D | TNF receptor superfamily member 10d [Source:HGNC Symbol;Acc:HGNC:11907] | 10055 | -0.326 | -0.2074 | Yes |
| 91 | NCR1 | natural cytotoxicity triggering receptor 1 [Source:HGNC Symbol;Acc:HGNC:6731] | 10076 | -0.328 | -0.1985 | Yes |
| 92 | FYN | "FYN proto-oncogene, Src family tyrosine kinase [Source:HGNC Symbol;Acc:HGNC:4037]" | 10092 | -0.330 | -0.1891 | Yes |
| 93 | MAP2K1 | mitogen-activated protein kinase kinase 1 [Source:HGNC Symbol;Acc:HGNC:6840] | 10197 | -0.340 | -0.1864 | Yes |
| 94 | KRAS | "KRAS proto-oncogene, GTPase [Source:HGNC Symbol;Acc:HGNC:6407]" | 10544 | -0.377 | -0.2015 | Yes |
| 95 | PLCG1 | phospholipase C gamma 1 [Source:HGNC Symbol;Acc:HGNC:9065] | 10722 | -0.395 | -0.2027 | Yes |
| 96 | ULBP2 | UL16 binding protein 2 [Source:HGNC Symbol;Acc:HGNC:14894] | 10724 | -0.396 | -0.1902 | Yes |
| 97 | TNFRSF10C | TNF receptor superfamily member 10c [Source:HGNC Symbol;Acc:HGNC:11906] | 10731 | -0.397 | -0.1781 | Yes |
| 98 | IFNB1 | interferon beta 1 [Source:HGNC Symbol;Acc:HGNC:5434] | 10801 | -0.406 | -0.1705 | Yes |
| 99 | HLA-G | "major histocompatibility complex, class I, G [Source:HGNC Symbol;Acc:HGNC:4964]" | 10869 | -0.413 | -0.1626 | Yes |
| 100 | CHP1 | calcineurin like EF-hand protein 1 [Source:HGNC Symbol;Acc:HGNC:17433] | 10940 | -0.423 | -0.1546 | Yes |
| 101 | PIK3R2 | phosphoinositide-3-kinase regulatory subunit 2 [Source:HGNC Symbol;Acc:HGNC:8980] | 10956 | -0.425 | -0.1423 | Yes |
| 102 | TNFRSF10B | TNF receptor superfamily member 10b [Source:HGNC Symbol;Acc:HGNC:11905] | 11020 | -0.433 | -0.1334 | Yes |
| 103 | PIK3R1 | phosphoinositide-3-kinase regulatory subunit 1 [Source:HGNC Symbol;Acc:HGNC:8979] | 11099 | -0.443 | -0.1254 | Yes |
| 104 | KIR2DS3 | "killer cell immunoglobulin like receptor, two Ig domains and short cytoplasmic tail 3 [Source:HGNC Symbol;Acc:HGNC:6335]" | 11103 | -0.444 | -0.1115 | Yes |
| 105 | PTPN11 | protein tyrosine phosphatase non-receptor type 11 [Source:HGNC Symbol;Acc:HGNC:9644] | 11260 | -0.465 | -0.1089 | Yes |
| 106 | NCR3 | natural cytotoxicity triggering receptor 3 [Source:HGNC Symbol;Acc:HGNC:19077] | 11322 | -0.474 | -0.0985 | Yes |
| 107 | NFATC4 | nuclear factor of activated T cells 4 [Source:HGNC Symbol;Acc:HGNC:7778] | 11363 | -0.481 | -0.0864 | Yes |
| 108 | VAV2 | vav guanine nucleotide exchange factor 2 [Source:HGNC Symbol;Acc:HGNC:12658] | 11413 | -0.489 | -0.0746 | Yes |
| 109 | NCR2 | natural cytotoxicity triggering receptor 2 [Source:HGNC Symbol;Acc:HGNC:6732] | 11611 | -0.518 | -0.0736 | Yes |
| 110 | PTK2B | protein tyrosine kinase 2 beta [Source:HGNC Symbol;Acc:HGNC:9612] | 11618 | -0.520 | -0.0575 | Yes |
| 111 | HLA-E | "major histocompatibility complex, class I, E [Source:HGNC Symbol;Acc:HGNC:4962]" | 11659 | -0.526 | -0.0439 | Yes |
| 112 | KIR3DL1 | "killer cell immunoglobulin like receptor, three Ig domains and long cytoplasmic tail 1 [Source:HGNC Symbol;Acc:HGNC:6338]" | 11864 | -0.563 | -0.0419 | Yes |
| 113 | SOS1 | SOS Ras/Rac guanine nucleotide exchange factor 1 [Source:HGNC Symbol;Acc:HGNC:11187] | 12041 | -0.603 | -0.0364 | Yes |
| 114 | HLA-A | "major histocompatibility complex, class I, A [Source:HGNC Symbol;Acc:HGNC:4931]" | 12502 | -0.749 | -0.0486 | Yes |
| 115 | HLA-B | "major histocompatibility complex, class I, B [Source:HGNC Symbol;Acc:HGNC:4932]" | 12534 | -0.764 | -0.0267 | Yes |
| 116 | PPP3R1 | "protein phosphatase 3 regulatory subunit B, alpha [Source:HGNC Symbol;Acc:HGNC:9317]" | 12653 | -0.829 | -0.0095 | Yes |
| 117 | NFAT5 | nuclear factor of activated T cells 5 [Source:HGNC Symbol;Acc:HGNC:7774] | 12741 | -0.903 | 0.0124 | Yes |
Table: GSEA details [plain text format]

  

Fig 2: KEGG\_NATURAL\_KILLER\_CELL\_MEDIATED\_CYTOTOXICITY      
 Blue-Pink O' Gram in the Space of the Analyzed GeneSet

  

Fig 3: KEGG\_NATURAL\_KILLER\_CELL\_MEDIATED\_CYTOTOXICITY: Random ES distribution      
 Gene set null distribution of ES for **KEGG\_NATURAL\_KILLER\_CELL\_MEDIATED\_CYTOTOXICITY**
